# Clinical utility of plasma microbial cell-free DNA sequencing in determining microbiologic etiology of infectious syndromes in solid organ transplant recipients

**DOI:** 10.1177/20499361241308643

**Published:** 2024-12-23

**Authors:** Jesal R. Shah, Muhammad Rizwan Sohail, Todd Lasco, John A. Goss, Mayar Al Mohajer, Sarwat Khalil

**Affiliations:** Baylor College of Medicine, Department of Medicine, Section of Infectious Diseases, Houston, TX, USA; Baylor College of Medicine, Department of Medicine, Section of Infectious Diseases, Houston, TX, USA; Baylor College of Medicine, Department of Pathology and Immunology, Houston, TX, USA; Department of Pathology, Baylor St Luke’s Medical Center, Houston, TX, USA; Division of Abdominal Transplantation, Baylor College of Medicine, Houston, TX, USA; Baylor College of Medicine, Department of Medicine, Section of Infectious Diseases, Houston, TX, USA; Baylor College of Medicine, Department of Medicine, Section of Infectious Diseases, Houston, TX 77030-3498, USA

**Keywords:** cell free DNA, immunocompromized, next-generation sequencing, solid organ transplant

## Abstract

**Background:**

Metagenomic next-generation sequencing (mNGS) is increasingly being used for microbial detection in various infectious syndromes. However, data regarding the use of mNGS in solid organ transplant recipients (SOTR) are lacking.

**Objectives:**

To describe and analyze real-world clinical impact of mNGS using plasma microbial cell-free DNA (mcfDNA) in SOTR.

Design: Retrospectively reviewed all adult SOTR who underwent mNGS testing using plasma mcfDNA at Baylor St Luke’s Medical Center from March 2017 to February 2023.

**Methods:**

Clinical impact (positive, neutral, and negative) was assessed using standardized objective criteria. Three Infectious Diseases physicians independently performed clinical adjudication to determine the correlation of mcfDNA results with clinical diagnosis. A descriptive analysis of the patient and clinical characteristics was performed.

**Results:**

A total of 113 mcfDNA tests in liver (42%), kidney (35%), lung (20%) and heart (13%) transplant recipients were performed in the study period. The most common clinical syndromes were pneumonia (36%), fever of unknown origin (16%), and intra-abdominal infections (15%). Most (80, 71%) of the mcfDNA test results were positive for microorganisms. Twenty-seven (24%) cases were classified as positive clinical impact, 82 (73%) were neutral and 4 (3%) were negative, respectively.

**Conclusion:**

In SOTR, mcfDNA sequencing can add a positive clinical impact in a quarter of the cases and identify microorganisms beyond conventional microbiological testing across clinical syndromes. The negative clinical impact was rare. However, larger prospective studies are needed to define the optimal timing and utilization of mcfDNA in the sequence of diagnostic evaluation for syndrome-specific workup in SOTR.

**Summary:**

Metagenomic next-generation sequencing (mNGS) is a novel diagnostic tool that can identify difficult-to-detect microorganisms in SOTR. Our study demonstrates that the mNGS test resulted in a positive clinical impact in 1 out of 4 patients.

## Plain language summary

Use of plasma cell-free DNA testing to diagnose microbiologic causes of infections in solid organ transplant recipients

In our study, we evaluated the use of metagenomic next-generation sequencing (mNGS) in solid organ transplant patients who were hospitalized with concerns for infection that was difficult to diagnose by standard methods of testing. We reviewed 113 patients and found that the most common syndromes in which mNGS aided in diagnosis was pneumonia (36%), followed by fever of unknown origin (16%), and intra-abdominal infections (15%). The test result was found to be positive in 80 patient for at least one or more microorganisms. Based on the standardized clinical definitions to look at the impact of obtaining mNGS test, we found that the test had a positive clinic impact in 24% of the patients, neutral impact in 73%, and a minimal negative impact of 3%, respectively. This study shows that mNGS can be helpful in appropriately managing infections in one out of four patients with solid organ transplant.

## Introduction

Advancements in medical care and immunosuppression have significantly improved graft survival rates in solid organ transplant recipients (SOTR). However, due to their prolonged immunosuppressive state, SOTR face an increased risk of severe, life-threatening infections, both typical and opportunistic. Prompt diagnosis of infection in these high-risk patients is crucial for instituting appropriate antimicrobial therapy.

Conventional microbiologic testing (CMT), such as cultures, serologies, and pathogen-specific polymerase chain reactions (PCRs), may be negative in more than half of the cases.^1,2^ Prior antimicrobial therapy may further reduce the yield of cultures. Moreover, slow-growing and fastidious organisms may lead to delayed or missed diagnosis. Serological testing is frequently limited by low sensitivity and specificity. Multiple conventional PCRs are sometimes necessary to identify causative pathogens in the event of opportunistic or atypical infections.

When non-invasive CMT fails to provide a diagnosis, invasive procedures such as bronchoscopy and tissue biopsy may be required for the diagnosis of focal infections. These invasive tests carry risks of complications, causing reluctance or refusal from patients and providers to pursue tissue diagnosis. Consequently, empiric treatment is often employed, which may be either inadequate in covering the actual pathogen or excessively broad, leading to the development of resistance and an increased risk of adverse events. Thus, there is an urgent need for novel diagnostic methods to overcome the limitations of CMT.

Metagenomic next-generation sequencing (mNGS) is an emerging novel test that uses the sequencing of multiple DNA fragments. It offers exponentially faster sequencing and generates vast amounts of DNA sequences at substantially reduced costs. mNGS of plasma microbial cell-free DNA (mcfDNA) has the potential to avoid invasive procedures, optimize the time to diagnosis, and increase the diagnostic yield for fastidious organisms.^3^

Prior studies have evaluated the concordance of plasma mcfDNA with CMT in patients with febrile neutropenia, invasive fungal infections, infective endocarditis, fever of unknown origin (FUO), and other syndromes. Utility of the plasma mcfDNA for monitoring cytomegalovirus (CMV) infection and detecting other pathogens was assessed in a recent prospective study of hematopoietic stem cell transplant recipients.^4^ In this study, mcfDNA sequencing detected CMV with high accuracy and had a good correlation with traditional quantitative PCR. Moreover, mcfDNA testing detected pathogens earlier than CMT and those missed by CMT. Earlier studies have included some SOTR cases as part of the study population, but none have specifically evaluated the utility of plasma mcfDNA sequencing in this group of patients alone.^5–13^

Our study exclusively focuses on this subpopulation of patients to identify the real-world clinical impact of this technology in SOTR.

## Methods

We performed a retrospective review of all adult SOTR who underwent the Karius test at Baylor St Luke’s Medical Center, an 881-bed academic hospital in Houston, TX, from March 2017 to February 2023. Patients who were 18 years of age or older, who were SOTR, and who underwent mcfDNA testing were included. Patients who met the following criteria were excluded: Failed renal transplant and not on immunosuppression, or history of hematopoietic stem cell transplant in addition to solid organ transplant. The study was approved by the institutional review board at Baylor College of Medicine.

At our institution, mNGS ordering is limited to Infectious Diseases (ID) specialists. Indications for ordering mNGS are standardized and include culture-negative endocarditis, FUO, HIV/AIDS with fever, SOTR with fever over 48 h and negative workup, systemic and deep-seated infection where biopsy or CMT is negative, systemic and deep-seated infection where biopsy or other workup is not possible or not preferred, and other indications (as per ordering ID physician).

mNGS was done using Karius™ (Redwood City, CA, USA), which is a commercially available test of plasma mcfDNA that can identify over 1200 bacteria, DNA viruses, fungi, and eukaryotic parasites.^14^ All Karius tests were collected, stored, and sent to the reference lab. Specimens were processed with DNA extraction and library preparation, followed by mcfDNA sequencing and analysis with a curated clinical-grade pathogen database. Results were reported approximately 1 day after the specimen was received by the reference lab.

We conducted a descriptive analysis of demographic and baseline patient characteristics (Table 3), mNGS test details and results (Table 4), diagnostic yield of mNGS and CMT (Table 5), and clinical impact of mNGS (Table 6). Medians and interquartile range (IQR) were used for most patient and clinical variables due to a non-normally distributed patient population.

The time of onset of infection after transplant was divided into three categories: Early (<1 month post-transplant), Intermediate (1–6 months post-transplant), and Late (>6 months post-transplant).

A standardized objective criterion was utilized to determine the clinical impact (positive, neutral, and negative) using a modified version of definitions by Hogan et al. (Table 1). The clinical impact classification was determined by the primary investigator (JS).^15^ Any case classification that was deemed equivocal by JS was then subsequently reviewed by the senior investigator (SK) and classified with mutual agreement.

**Table 1.** Criteria for clinical impact of mNGS (Positive, Neutral, and Negative).*

Positive	Neutral	Negative
• mNGS enabled de-escalation of therapy • mNGS enabled initiation of appropriate therapy, including appropriate escalation of therapy • mNGS allowed avoidance of invasive interventions • mNGS confirmed clinical diagnosis • New diagnosis based on mNGS, not by conventional methods • Earlier diagnosis based on MNGS	• mNGS showed new organism, no change in management • mNGS confirmed conventional testing result/diagnosis, no change in management • mNGS with negative result, no change in management • Patient death before mNGS result • mNGS showed a new organism, no documented clinical decision in response to test result	• mNGS led to unnecessary treatment • mNGS led to unnecessary diagnostic evaluation • mNGS led to longer length of hospital stay

*Modified from criteria proposed by Hogan et al.^15^

mNGS, metagenomic next-generation sequencing.

Clinical adjudication was performed by three clinical ID physicians independently to determine the alignment of KT results with clinical diagnosis. Two physicians (JS and SK) independently reviewed charts of all patients with positive KT to determine whether KT results were in concordance with clinical diagnosis. In cases of discrepancy between the two physicians, a senior physician (MS) was consulted to reach a consensus. Clinical adjudication was defined as definite, probable, possible, and unlikely using a modified version of the criteria established by Benamu et al.^5^ These definitions are outlined in Table 2.

**Table 2.** Clinical adjudication criteria.

Definite	mNGS pathogen result is concordant with at least 1 pathogen identified on CMT performed and is likely consistent with clinical, radiologic, and/or lab diagnosis of infection.
Probable	mNGS and CMT results are discordant, but pathogen identified on mNGS is a likely cause of infection based on clinical, radiologic, or laboratory findings.
Possible	mNGS and CMT results are discordant. mNGS result is consistent with the infection but not a common cause, based on adjudicators of clinical experience and available literature in SOT recipients.
Unlikely	mNGS is positive and discordant with CMT and/or not a plausible cause of infection. There is a more likely explanation for the clinical diagnosis not meeting “Possible” classification criteria.

Modified from criteria proposed by Benamu et al.^5^

CMT, conventional microbiological testing; mNGS, metagenomic next-generation sequencing; SOT, solid organ transplant.

The study was conducted and reported in accordance with the STROBE statement.^16^

## Results

A total of 113 mNGS sent in liver (42%), kidney (35%), lung (20%), and heart (13%) adult transplant recipients were identified during the study period (Table 3). The study population’s median age was 59 years (45–67), and 72% were male.

**Table 3.** Demographic and patient characteristics (*N* = 113).

Median age (Year) (IQR)	59 (45–67)
No. (%) of Female	32 (28%)
No. (%) of Male	81 (72%)
No. (%) of solid organ transplant type	
Heart	15 (13%)
Lung	22 (20%)
Liver	47 (42%)
Kidney	39 (35%)
Other	2 (2%)
No. (%) with comorbid conditions	
Hypertension	62 (58%)
Chronic kidney disease	62 (58%)
Type 2 diabetes mellitus	45 (42%)
Heart failure	31 (29%)
Coronary artery disease	27 (25%)
COPD/Other advanced lung disease	14 (13%)
Cerebral vascular accidents	13 (12%)
Others (Autoimmune diseases, HIV, Vascular Grafts, Prosthetic Joints, mechanical cardiac devices, and prosthetic valves)	9 (8%)

COPD, chronic obstructive pulmonary disease; HIV, human immunodeficiency virus; IQR, interquartile range.

**Figure 1.**
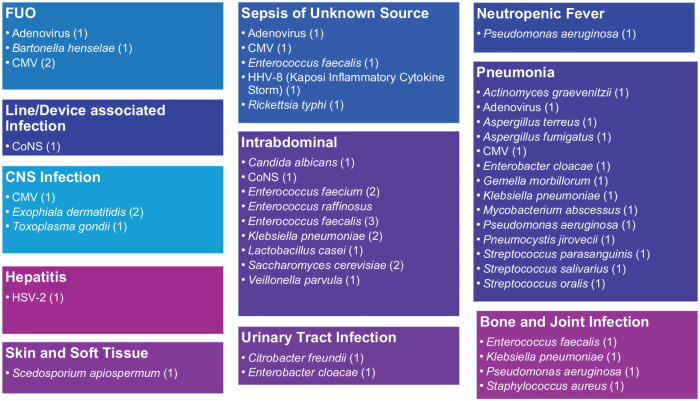
Definite and probable cases based on clinical adjudication of positive mNGS results (stratified by clinical syndrome). (Number of isolates in parenthesis). CMV, Cytomegalovirus; CNS, Central Nervous System; CoNS, Coagulase-negative Staphylococcus; FUO, Fever of Unknown Origin; HSV, Herpes Simplex Virus; HHV, Human Herpes Virus; mNGS, metagenomic next-generation sequencing.

The most common indication for mNGS use, based on the review of medical documentation, was to establish diagnosis (92%), followed by rule-out of infection (17%) (Table 4). The top-cited clinical syndromes of concern were pneumonia (36%), FUO (16%), and intra-abdominal infections (15%). Overall, 80 (71%) of the mNGS results detected an organism; 34 (43%) tests were positive with one organism, and 46 (58%) positive with >2 organisms. The specific organisms recovered from mNGS that were deemed definite and probable based on clinical adjudication are listed in Figure 1. The median turnaround time for mNGS results was 26 h, while the median time to reach a clinical decision after sending mNGS testing was 4 days. Of note, no clinical decision or action was documented in the patient chart for 12 mNGS results.

**Table 4.** mNGS test details and results.

Time to Result for mNGS (Median Hours) (IQR)	26 (25–27)
Time to clinical decision after mNGS sent (Median days, range)	4 (3–5)
No. (%) with No response	13 (12%)
No. (%) of Positive mNGS result	80 (71%)
No. (%) of Negative mNGS result	33 (29%)
Positive mNGS results	
1 organism	34 (43%)
>2 organisms	46 (58%)
Median (IQR) No. (Days) of antimicrobial therapy for clinical syndrome of concern (before mNGS sent)	
Antibacterial	6 (2–12)
Antifungal (Mold)	0 (0–4)
Antifungal (Yeast)	0 (0–9)
Antiviral	0 (0–0)
Previously on active antimicrobial agent for positive mNGS result (%)	
Yes	54%
No	46%
Indications for mNGS use	
Diagnosis	104 (92%)
Rule-out infection	19 (17%)
Avoid invasive procedure	10 (9%)
Monitoring of infection	8 (7%)
Other	1 (1%)
Clinical syndrome of concern	
Pneumonia	41 (36%)
Fever of unknown origin	18 (16%)
Intrabdominal infection (Abscess, Colitis, Cholangitis)	17 (15%)
CNS infection	14 (12%)
Unexplained leukocytosis	14 (12%)
Sepsis of unknown source	12 (11%)
Bone/Joint Infection	5 (4%)
Skin and soft tissue infections	5 (4%)
Bacteremia of unknown source	3 (3%)
Urinary tract infection	2 (2%)
Endocarditis	1 (1%)
Other	12 (11%)

CNS, central nervous system; IQR, interquartile range; mNGS, metagenomic next-generation sequencing; PCR, polymerase chain reaction.

Prior to mNGS being sent, patients were on a median of 6 days of antibacterial, 0 days of antifungal (mold or yeast), and 0 days of antiviral therapy for clinical syndrome of concern. In 54% of the cases, the patient was on an active antimicrobial agent for the positive mNGS result. Among the 113 mNGS sent in SOTR, 53 (47%) had positive CMT, with cultures as the predominant positive result.

From the standpoint of a positive recovery of a microbiological organism through the workup, 36 (33%) SOTR only had a positive mNGS result, 44 (38%) had both a positive mNGS and CMT result, and 9 (8%) only had a positive CMT result. A total of 24 (21%) of SOTR had both a negative mNGS and CMT result (Table 5).

**Table 5.** Diagnostic yield for mNGS and CMT results.

Positive CMT results	
Overall	53 (47%)
Culture	32
PCR	12
Serology	4
Biomarker	6
Antigen	1
Histopathology	10
Other	1
Diagnostic yield (No, %)	
Positive mNGS, Negative CMT	36 (33%)
Positive mNGS, Positive CMT	44 (38%)
Negative mNGS, Positive CMT	9 (8%)
Negative mNGS, Negative CMT	24 (21%)

CMT, conventional microbiologic testing; mNGS, metagenomic next-generation sequencing; PCR, polymerase chain reaction.

**Figure 2.**
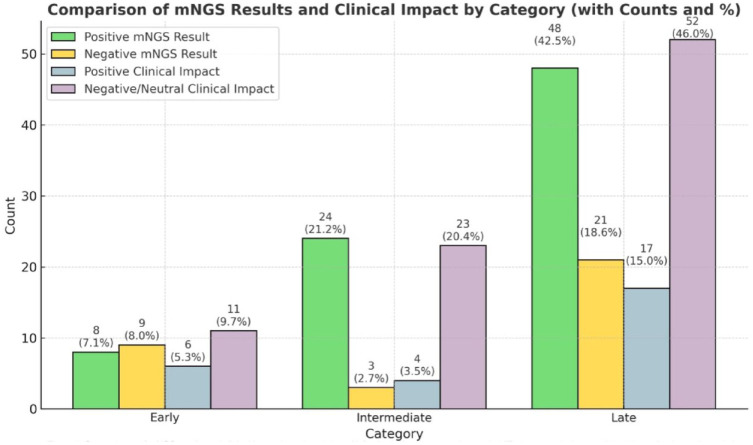
Comparisons of mNGS results and clinical impact based on timing of infection in the post-transplant period (early < 1 month, intermediate 1–6 months, late > 6 months). mNGS, metagenomic next-generation sequencing.

We assessed the microbiologic yield of mNGS testing and clinical impact trends categorized by the timing of infection in the post-transplant period. Positive mNGS results were more frequent in the late-onset infection category, indicating a higher detection rate of causative pathogens. Similarly, positive clinical impact on patient care (15%) was more common in late-onset infections (Figure 2).

Regarding clinical impact, 27 (24%) of mNGS results led to a positive clinical impact (Table 6); most commonly due to a new diagnosis (15, 56%) and change to appropriate therapy (14, 52%). The 24 cases with a positive clinical impact are further detailed in Supplemental Table S1. In four mNGS results, there was a negative clinical impact in the form of unnecessary treatment and diagnostic intervention. The majority (82, 73%) of mNGS results had a neutral clinical impact. This was mainly due to finding a new organism that led to no change in management (43, 52%): 26 of those 43 mNGS results were due to nonpathogenic organisms, five were related to treatment with the current regimen, and 12 from no documented clinical decision in response to the mNGS result. The second highest reason for neutral clinical impact was that the mNGS result had a negative result that led to no change in management (28, 34%).

**Table 6.** Clinical impact of mNGS results.

Positive impact, *N* = 27 (24%)	
Change to appropriate therapy	14 (52%)
De-escalation of therapy	9 (33%)
Avoidance of invasive procedure	3 (11%)
New diagnosis	15 (56%)
Earlier diagnosis	7 (26%)
Confirmed clinical diagnosis	2 (7%)
Negative impact, *N* = 4 (3%)	
Change in management	
Unnecessary treatment	2 (50%)
Unnecessary diagnostic intervention	2 (50%)
Increased LOS	0 (0%)
Neutral impact, *N* = 82 (73%)	
New organism, no change in management	43 (52%)
Nonpathogenic	26/43
Treated with current regimen	5/43
No documented clinical decision in response to mNGS result	12/43
Confirmed CMT result/diagnosis, no change in management	14 (17%)
Negative mNGS result, no change in management	28 (34%)
Patient death before mNGS result	0 (0%)

* Some cases were categorized into multiple subcategories of positive or neutral impact. For example, a single patient could have both an earlier diagnosis and a change in therapy. As a result, the cumulative percentages may exceed 100%.

CMT, conventional microbiologic testing; LOS, length of stay; mNGS, metagenomic next-generation sequencing.

All clinical cases with positive mNGS were adjudicated based on definitions provided in Table 2. Cases were classified as definite 25, probable 11, possible 6, and unlikely 38. Distribution of microorganisms identified for definite and probable cases for various clinical syndromes, based on clinical adjudication of mNGS, are summarized in Figure 1.

## Discussion

In this study, we examined the real-world utility of mNGS in the largest SOTR cohort to date. Our analysis shows that mNGS resulted in a one-third increase in diagnostic yield in the form of a positive microbiological result compared to CMT. Moreover, very few cases (8%) had a negative mNGS test in the setting of a positive CMT. We found that mNGS had a positive clinical impact in approximately 1 of 4 SOTR. The negative clinical impact was rare and observed in only 3% of the cases. These findings suggest that mNGS is a helpful tool to increase microbiologic detection without missing positive results typically detected with CMT.

Published data on the utility and clinical impact of plasma mcfDNA for detecting clinically significant pathogens are highly variable and appear to differ across infectious syndromes and patient populations. Earlier studies, such as Hogan et al.,^15^ which examined the clinical impact of mNGS in both immunocompetent and immunocompromized pediatric and adult populations, reported a low positive clinical impact of only 7.3% and a negative clinical impact of 3.7%. In contrast, a more recent retrospective review from our institution, focusing on FUO in immunocompetent adults, showed a significant positive clinical impact of 40% with minimal negative clinical impact (2.8%).^8^ This difference suggests that the sensitivity, specificity, and clinical utility of plasma mcfDNA likely depends on multiple factors, including pretest probability, patient populations (immunocompetent vs immunocompromized), and the clinical syndrome of interest. This is further supported by a recent prospective multicenter observational study to identify the etiology of pneumonia using bronchoscopy comparing the diagnostic yield of CMT and mNGS in immunocompromized adults, which showed that plasma mcfDNA had an additive diagnostic value of 12.1% (*p* < 0.001). Furthermore, in a subgroup analysis of their patients with negative CMT, mNGS had an additive diagnostic value of 17.4%.^17^ In another study, utility of mNGS was assessed in diagnosing endocarditis in patients with prior antimicrobial therapy by serial testing during admission.^18^ Investigators reported that plasma mcfDNA sequencing was able to detect causative pathogens for a much longer duration compared to blood cultures (median 38.1 days vs 3.7 days, respectively) after initiation of antibiotic therapy.

No prior studies have specifically evaluated mNGS in SOTR. However, in earlier studies that included both immunocompromized and immunocompetent patients, the positive clinical impact ranged from 7% to 56%.^8,10,12,15^ A single-center retrospective cohort study of 80 adult patients that included 21 SOTR by Shishido et al.^10^ noted that positive clinical impact was highest in SOTR (71.4%) and in patients who had been on antimicrobial therapy for shorter duration. In our study, the median duration of antimicrobial therapy prior to mNGS testing for clinical syndromes of concern was 6 days (IQR 2–12 days), which could have resulted in a relatively lower positive clinical impact observed in 1 of 4 patients. Moreover, we reviewed the clinicians’ response to mNGS (based on electronic medical records, EMR, and documentation) and found that the median time to act upon test results was 4 days despite a turnaround time of 26 h. There is a potential that earlier ordering of mNGS and more prompt incorporation of the results in clinical decision-making could lead to even greater positive clinical impact in the form of avoiding invasive procedures, earlier diagnoses, and fewer negative mNGS test results. This should be an area for future prospective investigations.

Few other published case reports and series highlight the potential applications of mNGS for detecting opportunistic infections using plasma mcfDNA in SOTR, such as diagnosing *Pneumocystis jirovecii* pneumonia (PJP) in renal transplant recipients.^11,19^ In our study, opportunistic infections detected by mNGS included *Bartonella henselae*, PJP, *Aspergillus* spp., *Saccharomyces* spp., *Scedosporium* spp., *Toxoplasma gondii*, and *Rickettsia typhi*, among others (Figure 1), that may be missed on CMT.

We adjudicated all clinical cases with positive mNGS results in our study cohort using standardized definitions. Among the cases, 25 were classified as definite, 11 as probable, 6 as possible, and 38 as unlikely. The relatively high proportion of cases categorized as “unlikely” underscores the critical role of infectious disease specialists in interpreting mNGS results. Their expertise is essential in integrating these results with clinical presentation, patient history, and other diagnostic findings to accurately differentiate true infections from incidental or nonpathogenic findings.

In our patient cohort, some cases had multiple organisms detected on mcfDNA or CMT. Only those organism(s) that aligned with the clinical diagnosis were deemed pathogenic, and any additional microorganisms found on either conventional or mNGS testing were designated as commensals or normal microbiota by the treating ID physicians.

Given the limited published data regarding the use of mNGS testing in SOTR, providing specific guidance for incorporating this testing strategy into routine clinical practice remains difficult. Nevertheless, mNGS shows promise in the evaluation of late-onset opportunistic infections, invasive fungal infections, and cases where traditional cultures yield negative results due to prior antimicrobial therapy or infections caused by difficult-to-culture or non-culturable organisms. In our cohort, mNGS resulted in higher microbiologic yield in the evaluation of late-onset infectious complications (especially after 6 months after organ transplant). However, clinicians should be mindful that despite the ability of mNGS to detect a wide range of typical and atypical pathogens, a negative mNGS result alone is not sufficient to exclude an infectious complication in SOTR. Similar to other molecular diagnostics, mNGS results must be interpreted within the broader clinical context.

Our study is limited by its retrospective design and smaller sample size and, therefore, is not powered to detect statistically significant differences in the performance of mNGS versus CMT. The clinical impact analysis depended on the subjective review and documentation of prior ID physicians’ notes. Non-EMR communication between the transplant team, microbiology lab, and ID consultants (which could affect clinical impact) was not captured. Retrospective clinical adjudication was challenging, as it was difficult to determine if the mNGS results aligned with clinical diagnosis. We attempted to mitigate this by having three ID physicians independently adjudicate the cases by classifying them into definite, probable, possible, and unlikely using standardized clinical definitions.

We assessed the microbiologic yield of mNGS results and clinical impact trends categorized by the timing of infection in the post-transplant period. Positive mNGS results and resultant positive clinical impact on patient care were more frequent in the late-onset infections category. However, due to small number of cases with positive clinical impact in each sub-category, we did not have the statistical power to do further subgroup analysis.

Assessing the overall healthcare cost implications of mNGS testing is complex. While earlier implementation of mNGS testing may enhance diagnostic accuracy, expedite the initiation of appropriate antimicrobial therapy, and potentially reduce the need for invasive procedures; the broader cost impact on both the healthcare system cannot be fully determined by the cost of mNGS testing alone. Aforementioned benefits of mNGS can potentially offset the upfront expense of mNGS testing. However, our study was not designed to evaluate these economic considerations.

Karius mNGS test itself had several limitations, including the need for processing at a specialized reference lab, resulting in a prolonged turnaround time. Additionally, the lack of standardization in quantifying the isolated pathogens in molecules per microliter in the earlier study period in our database hindered the investigation of clinical correlation.

## Conclusion

Our study findings demonstrate that mNGS positively impacted 1 out of 4 adult SOTR cases and identified microorganisms beyond CMT across clinical syndromes. However, clinical impact may vary based on pretest probability, the timing of infection onset in the post-transplant period, and an institution’s specific diagnostic stewardship policies. Careful review by ID specialists can help reduce inappropriate interpretations and unnecessary treatment or interventions in SOTR. Larger prospective studies are needed to define the optimal timing and utilization of diagnostic algorithms for syndrome-specific workup in SOTR.

## Supplemental Material

sj-docx-1-tai-10.1177_20499361241308643 – Supplemental material for Clinical utility of plasma microbial cell-free DNA sequencing in determining microbiologic etiology of infectious syndromes in solid organ transplant recipients Supplemental material, sj-docx-1-tai-10.1177_20499361241308643 for Clinical utility of plasma microbial cell-free DNA sequencing in determining microbiologic etiology of infectious syndromes in solid organ transplant recipients by Jesal R. Shah, Muhammad Rizwan Sohail, Todd Lasco, John A. Goss, Mayar Al Mohajer and Sarwat Khalil in Therapeutic Advances in Infectious Disease

## Declarations

**Ethics approval and consent to participate:** IRB approval from Baylor’s online Biomedical Research and Assurance Information Network (BRAIN) was obtained for this study (Ethics approval number: H-45143). As per the IRB protocol, informed consent was waived.

**Consent for publication:** Not applicable

**Availability of data and materials:** Not applicable.
