# Supplementary material for: Clinical utility of plasma microbial cell-free DNA sequencing in determining microbiologic etiology of infectious syndromes in solid organ transplant recipients
Source: Ther Adv Infect Dis. 2024 Dec 23;11:20499361241308643. doi: 10.1177/20499361241308643 (PMC11664510; doi:10.1177/20499361241308643)
Supplement: sj-docx-1-tai-10.1177_20499361241308643 – Supplemental material for Clinical utility of plasma microbial cell-free DNA sequencing in determining microbiologic etiology of infectious syndromes in solid organ transplant recipients [file sj-docx-1-tai-10.1177_20499361241308643.docx]

**Supplementary Tables:**

| **Table S1.** Summary table of demographic and clinical data for patients with positive clinical impact from mNGS. | | | | | | | | | | |
| --- | --- | --- | --- | --- | --- | --- | --- | --- | --- | --- |
| **Age/Gender** | **Type of SOT** | **Clinical Syndrome** | **Final Clinical Diagnosis** | **CMT Results** | mNGS **Results (Before or After CMT Result)** | **Time to Clinical Response after** mNGS **sent (days)** | **Positive Clinical Impact** | **Antimicrobial Therapy (days)** | **Prophylactic Therapy** | **Comments** |
| 61/Male | Kidney | Fever of unknown origin (FUO) | FUO secondary to *Bartonella henselae* | *Bartonella henselae* IgG 1:152 | ***Bartonella henselae***, Torque teno virus (BEFORE) | 11* | - New diagnosis, not by conventional methods** - mNGS enabled initiation of appropriate therapy | 0 | TMP-SMX | *Delay to result due to lab error  ** mNGS result prompted ordering of Bartonella serology to confirm diagnosis |
| 68/Male | Kidney | Pneumonia | Pneumonia secondary to *Aspergillus terreus* | Serum Galactomannan Ag (0.8), Repeat Serum Galactomannan Ag (1.8) | ***Aspergillus terreus***, *Mycoplasma hominis*, *Bacillus cereus, Bacillus thuringiensis, Ochrobactrum intermedium*, *Rhodococcus erythropolis*, Torque teno virus, Torque teno virus 1, Torque teno virus 27, Torque teno virus 28 (AFTER) | 3 | - New Diagnosis based on mNGS, not by conventional methods* | 9 (Antibacterial), 6 (*Candida*-Antifungal) | TMP-SMX, Valganciclovir | *Voriconazole started prior to result of mNGS, but mNGS provided species level information for new diagnosis |
| 23/Female | Liver | Cholangitis (Recurrent) | Cholangitis secondary to *Enterococcus* species | Negative | ***Enterococcus faecium, Enterococcus raffinosus*** (BEFORE) | 3 | - New Diagnosis based on mNGS, not by conventional methods - mNGS enabled de-escalation of therapy - mNGS enabled initiation of appropriate therapy | 2 (Antibacterial) | None |  |
| 55/Male | Liver | Pneumonia | Line infection related to *Enterobacter cloacae* and CoNS; Resolving *Fusarium* pneumonia | Negative | ***Enterobacter cloacae*, CoNS**, CMV, *Pseudomonas* spp., *Streptococcus mitis* (BEFORE) | 7 | - New Diagnosis based on mNGS, not by conventional methods* - mNGS enabled de-escalation of therapy** | 165 (C*andida* and Mold-Antifungal) | Atovaquone, Nystatin | *Result of *Enterobacter cloacae*, CoNS led to change in HD catheter ** mNGS with no *Fusarium* spp. (previously noted in BAL) 🡪 Completed 6 months of amphotericin therapy |
| 25/Female | Lung | Pneumonia | Pneumonia secondary to *Klebsiella pneumoniae* and *Aspergillus fumigatus* | Serum Beta-D-Glucan >500 | ***Klebsiella pneumoniae, Aspergillus fumigatus*** (AFTER) | 2 | - New Diagnosis based on mNGS, not by conventional methods - mNGS allowed avoidance of invasive interventions* | 6 (Antibacterial), 6 (*Candida* and Mold-Antifunga) | TMP-SMX, Valganciclovir | *Avoided repeat bronchoscopy  **Added ciprofloxacin on discharge for *Klebsiella pneumoniae* |
| 70/Male | Heart, Kidney | Sepsis of Unknown Source, Intrabdominal Infection | HSV-2 Hepatitis; Gut translocation with the following organism: *Saccharomyces cervevisiae, Klebsiella pneumoniae*, *Candida albicans, Candida clostroides, Veillonella parvula* | Negative | **HSV-2, *Saccharomyces cerevisiae, Klebsiella pneumoniae, Enterococcus faecalis, Candida albicans*, *Clostridiodes difficile*, *Veillonella parvula***  (BEFORE) | 4 | - New Diagnosis based on mNGS, not by conventional methods - mNGS allowed avoidance of invasive interventions - mNGS enabled initiation of appropriate therapy* - mNGS confirmed clinical diagnosis** | 10 (Antibacterial), 2 (Mold-Antifungal), 10 (*Candida*-Antifungal) | None | *Acyclovir for possible HSV-2 hepatitis  **Gut translocation vs Fistula in setting of recent small bowel resection with ileostomy formation |
| 62/Female | Liver | Unexplained Leukocytosis | GI perforation complicate by intrabdominal abscesses with Carbapenem Resistant *Klebsiella pneumoniae* and *Candida dubliniensis* | Surgical wound cultures | ***Klebsiella pneumoniae*** (BEFORE) | 4 | - Earlier Diagnosis based on   mNGS* | 12 (Antibacterial), 12 (*Candida*-Antifungal) | Ganciclovir, TMP-SMX | *Final Clinical Diagnosis: GI perforation, surgical cultures with Carbapenem-resistant *Klebsiella pneumoniae*, *Candida dubliniensis* |
| 34/Male | Liver | Sepsis of unknown source, unexplained leukocytosis | Acute cellular rejection of liver; sepsis secondary to *Sphigmonas* spp. infection | Tissue Pathology – Acute Cellular Rejection | **Positive** (low signal – *Sphigomonas* spp.) (AFTER) | 2 | - New Diagnosis based on mNGS, not by conventional methods* - mNGS enabled initiation of appropriate therapy* | 10 (Antibacterial), 10 (*Candida*-Antifungal) | Ganciclovir, Pentamidine | *Added amikacin as patient had been on carbapenem when mNGS resulted |
| 28/Female | Kidney* | Recurrent Pleural Effusion | Recurrent pleural effusion secondary to non-infectious etiology | Negative | **Negative** (AFTER) | 2 | - mNGS enabled de-escalation of therapy | 3 (Antibacterial), 3 (*Candida* – Antifungal) | Valganciclovir, TMP-SMX | *3 total kidney transplants |
| 63/Male | Heart | Skin and Soft Tissue Infection | Disseminated *Scedosporium apiospermum* infection | Tissue culture: *Scedosporium apiospermum* | BK polyomavirus, ***Scedosporium apiospermum*** (BEFORE) | 3 | - Earlier Diagnosis based on mNGS - mNGS enabled initiation of appropriate therapy - mNGS confirmed clinical diagnosis | None | None |  |
| 72/Female | Kidney | FUO | *Enterococcus faecalis* infection (unknown source) | Negative | ***Enterococcus faecalis**** (BEFORE) | 2 | - New Diagnosis based on mNGS, not by conventional methods - mNGS enabled initiation of appropriate therapy | 5 (Antibacterial) | None | *Unknown source, ID team recommend treatment |
| 55/Male | Kidney | Pneumonia, CNS Infection | Polymicrobial Aspiration pneumonia (with bolded organisms) | Negative | *Lactobacillus gasseri*, ***Streptococcus parasanguinis, Actinomyces graevenitzii***, *Lactobacillus fermentum,* ***Streptococcus salivarius* (*Streptococcus salivarus* group), *Streptococcus oralis* (*Streptococcus mitis group*)** (BEFORE) | 2 | - New Diagnosis based on mNGS, not by conventional methods - mNGS enabled de-escalation of therapy* | 4 (Antibacterial), 4 (Mold-Antifungal), 3 (*Candida*-Antifungal), 3 (Antiviral) | Valganciclovir, Pentamidine, Clotrimazole | *In conjunction with negative meningoencephalitis panel, ID team recommended stopping amphotericin and ganciclovir based on these results. Continued meropenem for pneumonia. |
| 64/Male | Liver | Diarrhea, Sepsis of Unclear Source | Disseminated *Saccharomyces cerevisiae* infection | Fungal blood culture: *Saccharomyces cerevisiae* | ***Saccharomyces cerevisiae*** (BEFORE) | 3 | - Earlier Diagnosis based on mNGS* | 12 (Antibacterial), 4 (*Candida*-Antifungal) | Aciclovir, TMP-SMX | *ID team recommend fungal blood cultures after mNGS result. No management change until fungal blood cultures reported positive as mNGS result was <10 MPM. |
| 64/Male | Liver | Disseminated Adenovirus, GVHD of the Intestine, History of Fungemia | Disseminated adenovirus; GVHD of the intestine with gut translocation (CoNS, *Enterococcus faecalis*) | Serum Adenovirus PCR positive | **Human Adenovirus C**, Torque teno virus**,** (AFTER)**;** **CoNS, *Enterococcus faecalis*** (BEFORE) | 2 | - New Diagnosis based on mNGS, not by conventional methods* - mNGS enabled initiation of appropriate therapy* | 60 (Antibacterial), 10 (*Candida*-Antifungal), 10 (Antiviral) | Ganciclovir, Atovaquone | *ID team started linezolid for possible gut translocation (*Enterococcus faecalis*, CoNS. Continued on existing therapy for known disseminated Adenovirus. |
| 65/Male | Liver | Pneumonia | Hypoxic respiratory failure secondary to pulmonary edema | Negative | **Negative** (AFTER) | 3 | - mNGS enabled de-escalation of therapy - mNGS allowed avoidance of invasive interventions | 27 (Antibacterial), 1 (*Candida* – Antifungal) | None |  |
| 67/Male | Heart | CNS Infection | Toxoplasma encephalitis | Serology: Toxoplasma IgM positive* | Torque teno virus 6, ***Toxoplasma gondii***, *Enterococcus faecalis*, Varicella-zoster virus (VZV), *Staphylococcus epidermidis*, *Rothia mucilaginosa*, CMV, *Lactobacillus gasseri* (BEFORE) | 2 | - New Diagnosis based on mNGS, not by conventional methods - Earlier Diagnosis based on mNGS - mNGS enabled initiation of appropriate therapy | 2 (Antibacterial) | None | *CNS Toxoplasma PCR negative (1 day prior to mNGS) |
| 49/Male | Liver | Neutropenic Fever | *Pseudomonas aeruginosa* bacteremia | Blood culture positive for *pseudomonas aeruginosa* | *Enterococcus faecalis,* ***Pseudomonas aeruginosa*,** CMV (BEFORE) | 3 | - Earlier Diagnosis based on mNGS | 27 (Antibacterial), 10 (*Candida*-Antifungal), 30 (Antiviral) | Pentamidine, Tenofovir |  |
| 49/Male | Liver | Pneumonia, Bacteremia of Unknown Source | VRE bacteremia; Pseudomonas aeruginosa pneumonia; CMV viremia | Blood Culture with MDR Pseudomonas, *Vancomycin-resistant Enterococcus faecium;* Sputum Culture with MDR *Pseudomonas aeruginosa*, CMV PCR Positive (1140) | *Enterococcus faecalis,* ***Pseudomonas aeruginosa,*** ***Enterococcus faecium*, CMV,** Herpes simplex virus type 1 (HSV-1) (AFTER) | 3 | - mNGS enabled de-escalation of therapy* | 45 (Antibacterial), 7 (Mold-Antifungal), 45 (*Candida*-Antifungal) | Tenofovir | *De-escalation of antifungal therapy. Continued treatment for known infections of VRE bacteremia, MDR *Pseudomonas* pneumonia/bacteremia and CMV viremia. |
| 33/Female | Lung | CNS Infection | CNS infection by *Exophiala dermatidis* | Serum Beta-D-glucan >500* | ***Exophiala dermatitidis*** (AFTER) | 3 | - New Diagnosis based on mNGS, not by conventional methods - mNGS enabled initiation of appropriate therapy | 4 (Antibacterial) | Valganciclovir, Atovaquone | *CSF: Cell count WBC 145 (PMN 100%) – fungal cultures with no growth |
| 40/Male | Liver, Kidney | Intrabdominal Infection (Peritonitis) | Spontaneous bacterial peritonitis | Negative | **Negative** (AFTER) | 3 | - mNGS enabled de-escalation of therapy | 3 (Antibacterial) | Valacyclovir | *Ascites cultures without growth |
| 43/Male | Kidney | CNS Infection, Sepsis of Unknown Source | Septic shock secondary to *Rickettsia typhi* | Negative | ***Rickettsia typhi*** (BEFORE) | 2 | - New Diagnosis based on mNGS, not by conventional methods - mNGS enabled initiation of appropriate therapy | 6 (Antibacterial) | TMP-SMX | *No rickettsial serologies performed |
| 53/Male | Heart | FUO | CMV infection | Serum CMV PCR Positive | **CMV** (BEFORE) | 6 | - Earlier Diagnosis based on mNGS - mNGS enabled initiation of appropriate therapy | 0 | TMP-SMX |  |
| 60/Female | Kidney, Pancreas | Pneumonia | Pneumonia secondary to *Pneumocystis jirovecii* | Negative | ***Pneumocystis jirovecii*,** *Bacteroides thetaiotaomicron* (*Bacteroides fragilis* group), *Phocaeicola vulgatus* (*Bacteroides vulgatus*) (*Bacteroides fragilis* group) (BEFORE) | 2 | - New Diagnosis based on mNGS, not by conventional methods** - mNGS enabled initiation of appropriate therapy | 46 (Antiviral) | None | *Bronchoscopy Biopsy/Cytology GMS stain negative **Hypoxic respiratory failure with diffuse ground glass opacities secondary to pneumocystis |
| 31/Male | Heart | Bone/Joint Infection | Lumbar osteomyelitis secondary to *Aspergillus fumigatus* | Serum beta-D-glucan >500* | ***Aspergillus fumigatus*** (BEFORE) | 3 | - New Diagnosis based on mNGS, not by conventional methods - mNGS enabled initiation of appropriate therapy | 42 (Antibacterial) | None | *L3-L4 disc aspiration cultures were negative |
| 45/Male | Liver | FUO, Intrabdominal Infection | Unknown | Negative | Negative (AFTER) | 3 | - mNGS enabled de-escalation of therapy | 8 (Antibacterial), 8 (*Candida*-antifungal) | Ganciclovir, TMP-SMX |  |
| 29/Female | Kidney | Pneumonia | CMV Pneumonitis | BAL CMV PCR 73,300 | **CMV**, *Pseudomonas aeruginosa*, *Pseudomonas fluorescens* (BEFORE) | 4 | - Earlier Diagnosis based on mNGS | 9 (Antibacterial), 9 (*Candida*-Antifungal), 9 (Mold-Antifungal) | TMP-SMX, Posaconazole | * mNGS resulted with CMV prompting ID team to request CMV PCR on subsequent BAL to work-up for possible CMV Pneumonitis |
| 50/Male | Liver | Unexplained Leukocytosis | Non-infectious physiological stressor leading to leukocytosis | Negative | Negative (AFTER) | 4 | - mNGS enabled de-escalation of therapy | 5 (Antibacterial), 10 (*Candida*- Antifungal) | TMP-SMX |  |

Abbreviations: CoNS, coagulase negative staphylococcus; CMT, conventional microbiologic testing; CMV, Cytomegalovirus; ID, infectious disease; FUO, fever of unknown origin; GVHD, graft versus host disease; HSV, Herpes Simplex Virus; mNGS, metagenomic next-generation sequencing; MDR, Multidrug resistant; PCR, polymerase chain reaction; PMN, polymorphonuclear leukocytes; WBC, white blood cell; TMP-SMX, trimethoprim (TMP) sulfamethoxazole (SMX); VRE, vancomycin resistant enterococci

**Bolded** organism on mNGS result is the organism thought to be clinically important.
